# Development of Instantly Dispersible Soy Protein Isolate Granules via Wet Granulation with HPMC and Crospovidone

**DOI:** 10.3390/foods15203600

**Published:** 2026-10-10

**Authors:** Pensak Jantrawut, Saroat Rawdkuen, Natthawuddhi Donlao, Wirongrong Tongdeesoontorn, Suphat Phongthai, Tanpong Chaiwarit

**Affiliations:** 1 Department of Pharmaceutical Sciences, Faculty of Pharmacy, Chiang Mai University, Chiang Mai 50200, Thailand; pensak.j@cmu.ac.th; 2 Center of Excellence in Agro Bio-Circular-Green Industry (Agro BCG), Faculty of Agro-Industry, Chiang Mai University, Chiang Mai 50100, Thailand; suphat.phongthai@cmu.ac.th; 3 Unit of Innovative Food Packaging and Biomaterials, School of Agro-Industry, Mae Fah Luang University, Chiang Rai 57100, Thailand; saroat@mfu.ac.th (S.R.); natthawuddhi.don@mfu.ac.th (N.D.); 4 School of Agro-Industry, Mae Fah Luang University, Chiang Rai 57100, Thailand; wirongrong.ton@mfu.ac.th; 5 Research Center of Innovative Food Packaging and Biomaterials Unit, Mae Fah Luang University, Chiang Rai 57100, Thailand; 6 Division of Food Science and Technology, Faculty of Agro-Industry, Chiang Mai University, Chiang Mai 50100, Thailand

**Keywords:** crosprovidone, granules, HPMC, reconstitution, soy protein isolate

## Abstract

Soy protein isolate (SPI) is a highly nutritious plant-based protein essential for preventing age-related conditions like sarcopenia; however, its application is severely hindered by inherent hydrophobicity and poor water dispersibility, which causes severe lumping. This study aimed to develop dispersible SPI granules by applying pharmaceutical excipients via wet granulation. SPI was granulated using hydroxypropyl methylcellulose (HPMC; 0.5–1.5%) as a binder and crospovidone (1.0–2.0%) as a superdisintegrant. The physicochemical and reconstitution properties were evaluated. The granulation process successfully transformed fine SPI powder into large, highly porous agglomerates. The optimized formulation (1.5% HPMC and 2.0% crospovidone) significantly reduced the dispersing time to 5.33 s and lowered the equilibrium contact angle (32.08°), promoting fast capillary water uptake. FTIR and XRD analyses confirmed the protein’s chemical and structural integrity remained unaltered, indicating purely physical interactions. DSC revealed a binder-concentration-dependent decrease in denaturation temperature due to plasticization. Furthermore, the reconstituted formulations exhibited shear-thinning rheological behavior. This study shows that the application of HPMC and crospovidone as modifying excipients in wet granulation presented a chemically inert granulation approach that overcomes hydrophobic lumping and improves plant protein dispersibility. This strategy highlights a strong potential for creating convenient nutritional supplements for the elderly.

## 1. Introduction

Proteins are crucial macronutrients that play a fundamental role in human health, contributing to muscle synthesis, tissue repair, and overall metabolic homeostasis [1,2]. Adequate dietary protein is particularly critical for the aging population to prevent several physiological conditions, such as sarcopenia, a progressive disorder characterized by the age-related loss of skeletal muscle mass and strength [3,4]. With the escalating global awareness of preventive nutrition, the demand for high-quality protein supplements has surged. Simultaneously, the global shift toward plant-based proteins aligns directly with the United Nations’ Sustainable Development Goals (SDGs 2, 3, and 12) by promoting sustainable agriculture and reducing the environmental footprint of animal farming [5]. Among plant-derived sources, soy protein isolate (SPI) stands out as a highly nutritious option. Structurally, SPI is a highly refined macromolecular complex containing protein, consisting predominantly of two major globular storage proteins: *β*-conglycinin (7S) and glycinin (11S). These specific globulins are largely responsible for the protein’s functional properties and its comprehensive amino acid profile [6].

Despite its outstanding nutritional profile, the application of SPI in powdered beverages is severely hindered by its poor macroscopic reconstitution properties and its high sensitivity to environmental conditions, particularly pH. The solubility, conformation, and functional integrity of SPI are strongly pH-dependent. Specifically, when the pH of the dispersing medium approaches the protein’s isoelectric point (approximately pH 4.5), the net charge of the soy globulins becomes zero, leading to severe intermolecular aggregation and minimal solubility [7]. Native SPI powders are characterized by extremely fine particle sizes and inherent hydrophobicity. When dispersed in water, they rapidly form a viscous, impermeable gel layer on the surface—a phenomenon known as “lumping” or “fish-eye” formation [8]. Consequently, achieving a homogeneous dispersion requires significant mechanical shear, necessitating vigorous shaking or prolonged stirring. For elderly consumers and individuals managing sarcopenia, this requisite physical exertion presents a substantial barrier to daily consumption, ultimately leading to poor dietary compliance.

Transforming the fine SPI powder into an agglomerated granular form presents a highly effective solution. While spray drying is the conventional method used to manufacture plant protein powders, it presents critical limitations specifically for SPI. The high inlet temperatures required in the spray drying process often trigger the irreversible thermal denaturation of the heat-sensitive 7S and 11S globulin fractions, severely compromising their innate solubility and functional integrity [9]. Furthermore, spray drying typically generates excessively fine, cohesive particles (<100 µm) that retain poor wetting characteristics and exacerbate the lumping phenomenon. To address these issues, recent research and industrial practices have actively explored various processing technologies to develop plant-based protein supplements. Common methodologies involve co-spray drying with carbohydrate carriers (e.g., maltodextrin) to enhance solubility, or the application of hydrophilic emulsifier coatings, such as lecithin, to improve surface wettability [10]. Additionally, particle enlargement techniques have been investigated; for instance, Dacanal and Menegalli successfully utilized fluidized bed agglomeration to restructure SPI into porous granules, demonstrating a significant improvement in powder wettability compared to the native powder [11]. In contrast, wet granulation offers distinct advantages for plant proteins by operating at milder temperatures, thereby preserving the structural integrity of the soy globulins. Moreover, it effectively binds fine powders into larger, highly porous agglomerates (>200 µm), which collectively facilitate rapid capillary water uptake and superior flowability [12,13].

This study introduces a high degree of novelty through a multidisciplinary approach, strategically applying advanced pharmaceutical technology to the field of food science. While conventional food-grade agglomeration typically relies on simple sugars, this research pioneers the combined application of pharmaceutical-grade excipients, for instance, hydroxypropyl methylcellulose (HPMC), a hydrophilic polymeric binder, and crospovidone, a highly potent superdisintegrant. Importantly, both HPMC and crospovidone possess well-established toxicological safety profiles. Both are Generally Recognized as Safe (GRAS) by food regulatory agencies and have been extensively utilized for decades as safe, inactive excipients in oral pharmaceutical solid dosage forms, particularly swallowable tablets [14]. Functionally, HPMC provides the necessary structural integrity and forms a hydrophilic film to reduce solid–liquid interfacial tension [15,16]. Conversely, crospovidone acts through an aggressive capillary wicking mechanism to facilitate ultra-fast water ingress and rapid granular disruption, entirely eliminating the need for vigorous mechanical shaking [17]. The combination of HPMC and crospovidone is particularly critical for overcoming the dense, hydrophobic nature of plant protein matrices. While HPMC acts as a polymeric binder that coats the particles to provide a highly hydrophilic surface—drastically lowering the solid–liquid interfacial tension during initial wetting [13,14]—crospovidone prevents the formation of a localized viscous gel block. Because crospovidone is a highly cross-linked, insoluble polymer, it does not dissolve to form a barrier; rather, it acts as a physical water conduit, drawing liquid deep into the granular core through aggressive capillary action [18]. In the context of nutraceuticals and food supplements, HPMC has been successfully utilized as a binder in wet granulation to combine hydrophilic vitamin B12 and hydrophobic vitamin D2 [19]. Furthermore, HPMC has proven to be an effective binder in *Cassia siamea* extract tablets [20]. Similarly, crospovidone, when used as a superdisintegrant in tablet formulations containing dry plant extracts, has demonstrated excellent pH-independent disintegration properties [21]. Another study reported that the formulation of herbal fast-disintegrating tablets utilizing crospovidone achieved exceptionally short wetting and disintegration times [22].

By merging pharmaceutical formulation strategies with nutritional science, this study aims to develop an instantly dispersible plant-based protein supplement and investigate the effects of varying concentrations of HPMC and crospovidone on the physicochemical, reconstitution, and rheological properties of SPI granules formulated with a complex base of hydrocolloids and flavoring agents.

## 2. Materials and Methods

### 2.1. Materials

Soy protein isolate (food grade) was purchased from ET-chem (Suzhou, China). Carrageenan (food grade) was purchased from Lab valley (Bangkok, Thailand). Maltodextrin was purchased from Himedia (Maharashtra, India). Crospovidone (Polyvinylpolypyrrolidone; PVPP, 5–50 µm, pharmaceutical grade) was purchased from TNJ Chemical (Hefei, China). Hydroxypropyl methylcellulose (HPMC) E5 was purchased from ONIMAX (Bangkok, Thailand). Sucralose, sodium saccharin and xanthan gum (food grade) were purchased from Krungthepchemi (Bangkok, Thailand). Cocoa powder 100% (10–12% fat content) was purchased from Sino-Pacific Trading (Bangkok, Thailand).

### 2.2. Soy Protein Isolate Formulation

Soy protein isolate and the other formulation excipients, including crospovidone, sucralose, maltodextrin, sodium saccharin, xanthan gum, carrageenan, and cocoa powder, were accurately weighed according to the composition presented in Table 1. The ingredients were then uniformly blended using the geometric dilution technique in a mortar and pestle. Upon achieving a homogeneous mixture, a 3% hydroxypropyl methylcellulose (HPMC) solution was added as a binder to facilitate particle agglomeration into larger granules. The resulting wet mass was passed through a No. 60 mesh sieve and subsequently dried for two cycles in a U204 fluid bed dryer (Sherwood Scientific, Cambridge, UK) at 50 °C for 15 min.

### 2.3. Particle Size

The method to measure particle size distribution was adapted from previous studies [13,23,24]. The particle size distribution was analyzed using a Mastersizer 3000 (Malvern Instruments Ltd., Malvern, UK). Approximately 15 g of each formulation was loaded into a feeding tray. Optical parameters for the analysis included refractive indices of 1.0 for the air dispersant and 1.45 for the particles. An obscuration range between 10% and 30% was maintained throughout the entire measurement duration. The particle size distribution was reported in terms of D [4, 3] (VMD, volume mean diameter), D [3, 2] (SMD, surface mean diameter), D (0.1), D (0.5), D (0.9), and Span. Each sample was measured five times.

### 2.4. Morphological Characteristics

The macroscopic morphological appearance of the formulated soy protein isolate (SPI) granules was initially evaluated by visual inspection. For microstructural analysis, the samples were mounted onto aluminum stubs using adhesive carbon tape and sputter-coated with gold for 1 min. The surface morphologies were observed using a scanning electron microscope (SEM; JEOL JCM-7000 NeoScope^TM^, Tokyo, Japan) at 100× magnification, operating under low-vacuum mode with an accelerating voltage of 15 kV.

### 2.5. FTIR Analysis

Fourier-transform infrared (FTIR) spectra were acquired in transmittance mode using an FT/IR-4700 spectrophotometer (Jasco Corp., Tokyo, Japan). The spectra were recorded across a wavenumber range of 4000 to 500 cm^−1^ with a spectral resolution of 4 cm^−1^.

### 2.6. Moisture Content

The method to determine moisture content was adapted from the previous study [25]. The analysis was conducted using an HC103 moisture analyzer (Mettler Toledo, Greifensee, Switzerland). Approximately 5 g of each sample was evenly distributed on a steel pan. The temperature was increased from room temperature to 200 °C at a heating rate of 10 °C/min. All measurements were performed in triplicate, and the final moisture content was calculated using Equation (1).

(1) $$\mathrm{Moisture}\;\mathrm{content}\;(\%)=\frac{\mathrm{Initial}\;\mathrm{mass}-\mathrm{dry}\;\mathrm{mass}}{\mathrm{Initial}\;\mathrm{mass}}\times \;100$$

### 2.7. Water Activity (a_w_)

The water activity of the samples was measured at a constant temperature of 25 °C using an AquaLab Series 3 TE water activity meter (Decagon Devices, Inc., Pullman, WA, USA). All measurements were performed in triplicate.

### 2.8. pH

The pH values were determined by dispersing 5 g of each formulation in 50 mL of deionized water. The pH of the resulting dispersions was measured at room temperature using a calibrated 691 pH meter equipped with a glass electrode (Metrohm, Herisau, Switzerland).

### 2.9. X-Rays Diffractometry (XRD)

The crystalline states of the SPI powder and formulations were analyzed using a Smartlab analytical X-ray diffractometer (Rigaku Corporation, Tokyo, Japan). The operating parameters included a voltage of 40 kV, a scanning rate of 10°/min, a step size of 0.01°, and a scanning range of 5° to 60°.

### 2.10. Thermal Properties

The thermal stability of the samples was analyzed using a differential scanning calorimeter (DSC1, Mettler Toledo, Columbus, OH, USA). Approximately 3–5 mg of each sample was accurately weighed and sealed inside an aluminum DSC pan. An empty sealed aluminum pan served as the reference. The pans were loaded into the analytical module and heated from 20 °C to 150 °C at a constant heating rate of 5 °C/min. Nitrogen was utilized as a purge gas at a flow rate of 30 mL/min to maintain an inert atmosphere during the measurements [26].

### 2.11. Water Reconstitution

#### 2.11.1. Dispersing Time

The method used to determine the wetting time of the formulations was modified from a previous study [27]. Wettability was assessed by determining the time required for the powder to completely submerge in water. Specifically, a 6 g powder sample was gently introduced onto the surface of 100 mL of distilled water at room temperature within a standard 400 mL beaker, under constant agitation at 500 rpm. This specific agitation rate was selected to ensure standardized measurement reproducibility, simulating continuous manual spoon-stirring rather than vigorous shaking [27,28]. The samples were classified as wettable if complete immersion occurred in under 60 s, and non-wettable if the process took longer than 120 s. All evaluations were performed in triplicate.

#### 2.11.2. Modified Washburn Method by Capillary Rise Wetting

Powder wettability was evaluated using a modified Washburn method, which characterizes capillary rise behavior by measuring the mass of the penetrating liquid rather than the traditional penetration depth [29]. Following a previously described method [27], a 2 g powder sample was loaded into a cylindrical glass tube equipped with an open base sealed by filter paper and gauze. The tube was suspended just above the surface of distilled water maintained at 25 °C. Wettability was then quantified by recording the total mass of water adsorbed by the powder bed over a 10-min period. The wettability was presented in terms of water adsorption, calculated using Equation (2). All measurements were performed in quintuplicate.

Water adsorption (g) = Final mass (g) − Initial mass (g)(2)

#### 2.11.3. Contact Angle

(1) Sessile drop method: Contact angle analysis provides a direct measurement of the tangent angle at the three-phase intersection. Because powder wetting is a dynamic phenomenon, wettability is best quantified by monitoring the rate at which this contact angle changes over time [8]. The formulation was compressed by a single-punch hydraulic machine (Carver Model C Laboratory Press, Fred S. Caver, Wabash, OH, USA) at a pressure of 1 ton to facilitate the loose aggregation of the granules and obtain a smooth surface. A 5 µL droplet of deionized water was deposited onto the leveled powder bed, and both the contact angle (θ) and the residual droplet volume above the solid phase were continuously tracked during the wetting process (10 s). A minimum of five replicates were conducted for each sample.
(2) Modified Washburn capillary rise method: The modified Washburn capillary rise method was adapted from [30]. The bottom of the capillary tube was sealed with filter paper. Subsequently, 0.5 g of the sample was packed into the tube by tapping at least 100 times. The prepared tube was gently lowered until its base made precise contact with the surface of purified water, which was contained in a beaker positioned on an analytical balance. The capillary penetration kinetics were evaluated by monitoring the mass loss of the test liquid as it wicked into the powder bed. Both the real-time mass data and penetration time were simultaneously captured using a digital camera focused on the balance display and a dedicated timer. *n*-Hexane was utilized as the perfectly wetting reference fluid. All measurements were performed in triplicate.

#### 2.11.4. Dispersibility Index (DI)

The dispersibility index (DI) represents the percentage of dry matter capable of passing through a sieve following brief manual agitation. The method used to determine the DI was based on a previously described study [27]. A 10 g powder sample was dispersed in 100 mL of deionized water (25 °C) within a 250 mL beaker. The mixture was stirred vigorously with a spatula for 15 s, completing exactly 25 circular motions across the beaker’s diameter. The resulting suspension was filtered through a 250 µm mesh sieve to isolate the dispersed fraction. The collected filtrate was then oven-dried overnight at 105 °C, and the remaining dry mass was weighed to calculate the DI using Equation (3). All measurements were performed in triplicate.

(3) $$\mathrm{DI}=\frac{W_{d}\;\times \;(100+w)}{W\;\times \;\frac{100\;-\;W_{m}}{100}}$$

where

w is the weight of sample (g);
W_d_ is the weight of the dry matter in the filtrate that passed through the 250 µm sieve (g);
W_m_ is the moisture content of the powder (%, *w*/*w*).

### 2.12. Apparent Viscosity

The method to evaluate apparent viscosity was adapted from [31]. To determine the apparent viscosity of the formulations, a Brookfield RVDV-II+ rotational viscometer (Brookfield Engineering Laboratories, Inc., Middleboro, MA, USA) equipped with a ULA spindle was utilized. Temperature was regulated using a HAAKE A81 circulating bath (Thermo Fisher Scientific, Waltham, MA, USA). Each formulation (5 g) was completely dispersed in deionized water (50 mL), filled into a cup-and-bob apparatus, and left to reach thermal equilibrium for 15 min. The sample temperature was strictly maintained at 25 ± 0.1 °C. Continuous shear measurements were performed by recording the shear stress as the shear rate ramped up from 0 to 100 s^−1^ (upward curve) and subsequently ramped down from 100 to 0 s^−1^ (downward curve). The flow curve data were modeled according to the Power Law equation, Equation (4). All samples were tested in triplicate.

(4) $$\eta =K\cdot {\left(\dot{\gamma }\right)}^{n-1}$$

where

*η* is the apparent viscosity (mPa.s);
$\dot{\gamma }$ is the shear rate ($s^{-1}$);
K is the flow consistency index ($\mathrm{Pa}\cdot s^{n}$);
*n* is the flow behavior index.

### 2.13. Total Protein Content

The total protein content was determined using the Dumas method, adapted from AOAC Official Method 992.23. For standard preparation, 0.20 g of the EDTA standard was weighed into tin foil, and this procedure was performed in ten replicates. For solid sample preparation, the formulations were first finely ground. Approximately 0.20 g of each ground sample was accurately weighed into a piece of tin foil, which was then tightly folded to encapsulate the powder. The prepared samples were introduced into the combustion furnace via an automatic sample loading system. Upon completion of the analysis, the processing software reported the results as the percentage of total nitrogen (% total nitrogen). The total protein content was subsequently calculated using a standard nitrogen-to-protein conversion factor (*F*) of 6.25, according to Equation (5).

(5) Protein (%)=Nitrogen (%)×F

### 2.14. Statistical Analysis

All experiments were performed in at least triplicate, and the data are expressed as the mean ± standard deviation (SD). Statistical analysis was performed using SPSS software (version 17; IBM Corporation, Armonk, NY, USA) to determine significant differences among the formulations. A one-way analysis of variance (ANOVA) was utilized for the statistical evaluations. A significance level of *p* < 0.05 was applied for all comparisons.

## 3. Results and Discussion

### 3.1. Soy Protein Isolate Formulation

**Figure 1:**
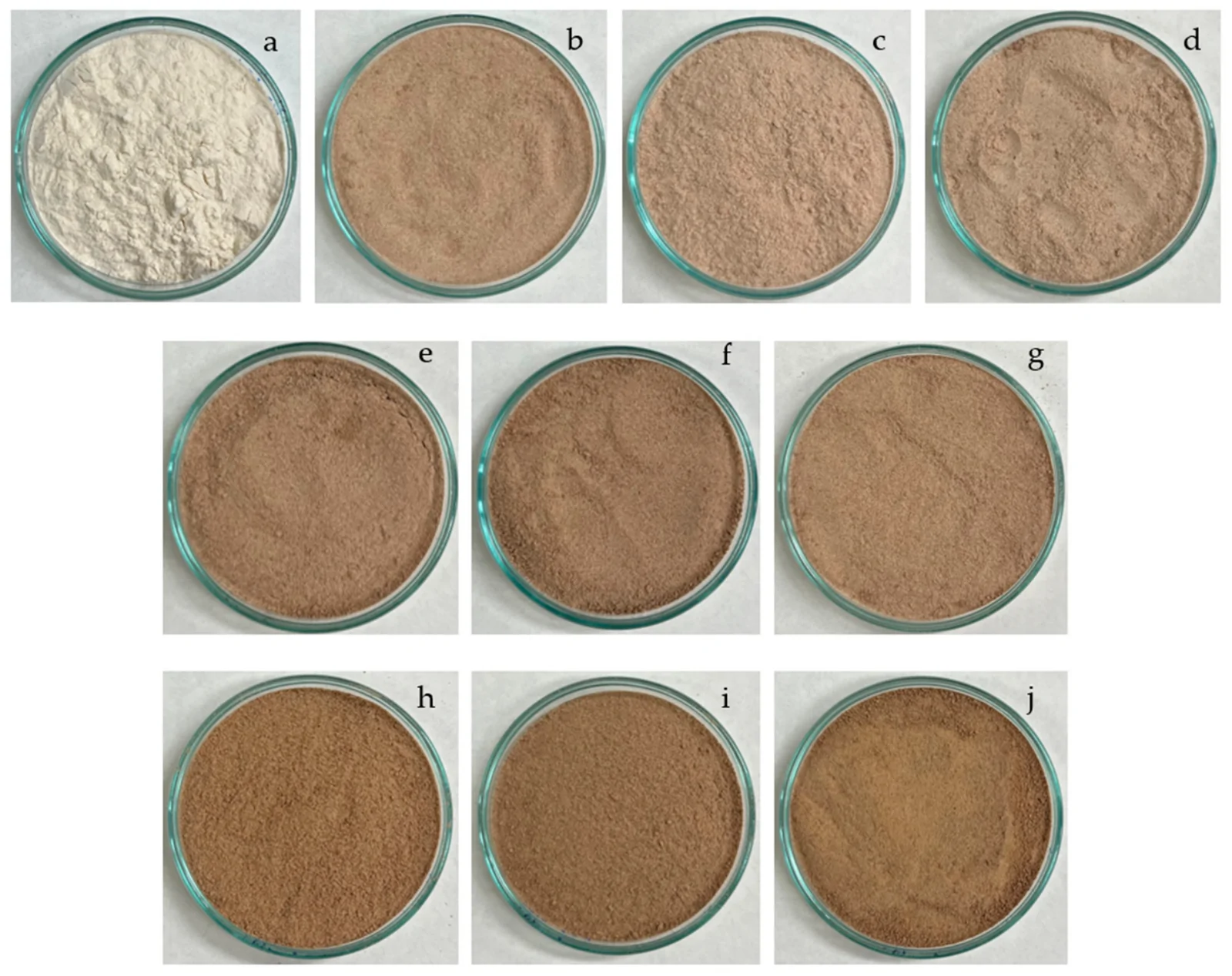
Visual appearance of SPI powder (**a**) and formulations 1–9 (**b**–**j**, respectively).

Appearance of SPI formulations is shown in Figure 1. The SPI formulations were prepared by varying the concentrations of hydroxypropyl methylcellulose (HPMC) solution as a binder (0.5%, 1.0%, and 1.5%) and crospovidone as a superdisintegrant (1%, 2%, and 3%). Visual inspection revealed distinct variations in color and granular size depending on the excipient ratios present in the formulations. The raw SPI consisted of a pale yellow powder, whereas the agglomerated formulations exhibited a brown color. Furthermore, the particle size visually tended to increase alongside an increasing ratio of the HPMC solution.

### 3.2. Particle Size

The particle size of the powder directly affects the physicochemical properties of plant-based protein supplements. The mean particle size was evaluated using both the Sauter mean diameter (D [3, 2]) and the volume mean diameter, also known as the De Brouckere mean diameter (D [4, 3]). D [3, 2] denotes the diameter of a sphere possessing the same volume-to-surface-area ratio as the entire sample distribution. D [4, 3] represents the volume- or mass-weighted average particle size, identifying the diameter at which the center of mass or volume of the particle distribution lies within a sample. The fine particle fraction was quantified using the D (0.1) metric, representing the diameter below which 10% of the total sample volume resides. D (0.9) and D (0.5) indicate the maximum particle diameters encompassing 90% and 50% of the total volume distribution, respectively. The span value represents the broadness of the size distribution [24,32].

The particle size distribution parameters—including D (0.1), D (0.5), D (0.9), D [3, 2], D [4, 3], and span—for the native SPI powder and Formulations 1–9 are presented in Table 2. The raw SPI powder exhibited a D [4, 3] of 280.0 ± 40.64 µm. Its broad particle size distribution, evidenced by a high span value of 19.55 ± 4.43, indicated significant size inconsistency, while a high D (0.9) value (1195.42 ± 301.76 µm) suggested the presence of large agglomerates. Furthermore, the D (0.1) of the native SPI powder was lower than that of all the prepared formulations, indicating a high fraction of fine particles, which are prone to cohesive aggregation. Together, these parameters confirmed that the native SPI powder possessed a highly heterogeneous particle size distribution. Such characteristics typically induce powder segregation during the mixing of other ingredients, ultimately adversely affecting the protein uniformity of the final product. Following the wet granulation process, which utilized a 3% HPMC solution as a polymeric binder, the span values of all formulations (1–9) were significantly reduced, ranging from 2.29 ± 0.19 to 3.69 ± 0.19. The concentration of the HPMC solution demonstrated a direct influence on the granule size. Formulations 1–3, which incorporated the lowest HPMC concentration (0.5%), produced granules with median diameters (D (0.5)) ranging from 71.86 ± 1.66 µm to 88.33 ± 5.17 µm. When the HPMC binder concentration was increased to 1.0% (Formulations 4–6) and 1.5% (Formulations 7–9), the D (0.5) values markedly shifted to higher ranges of 134.79 ± 1.73 µm to 163.67 ± 3.71 µm and 152.42 ± 3.17 µm to 180.76 ± 4.07 µm, respectively. Similar size-enlargement trends corresponding to the increase in HPMC concentration were consistently observed across the D [3, 2] and D [4, 3] parameters.

The prominent increase in the particle size parameters (D (0.1), D (0.5), D (0.9), D [3, 2], and D [4, 3]) corresponding to higher HPMC concentrations confirms the polymer’s robust efficacy as a wet binder. In wet granulation, higher binder concentrations provide increased viscosity and polymeric mass, facilitating the formation of stronger liquid bridges between the cohesive soy protein particles. This mechanism promotes greater granule coalescence and growth during the wet massing phase [33]. A previous study similarly found that increasing the HPMC binder concentration in the formulation produced larger, more porous, and irregularly shaped particles [34]. Conversely, crospovidone did not significantly alter the granule growth trajectory when its concentration was varied between 1% and 2%. Because crospovidone primarily functions through rapid water wicking and swelling without acting as a binding agent [35], it did not significantly interfere with the liquid bridge formation governed by HPMC.

### 3.3. Morphological Characteristics

**Figure 2:**
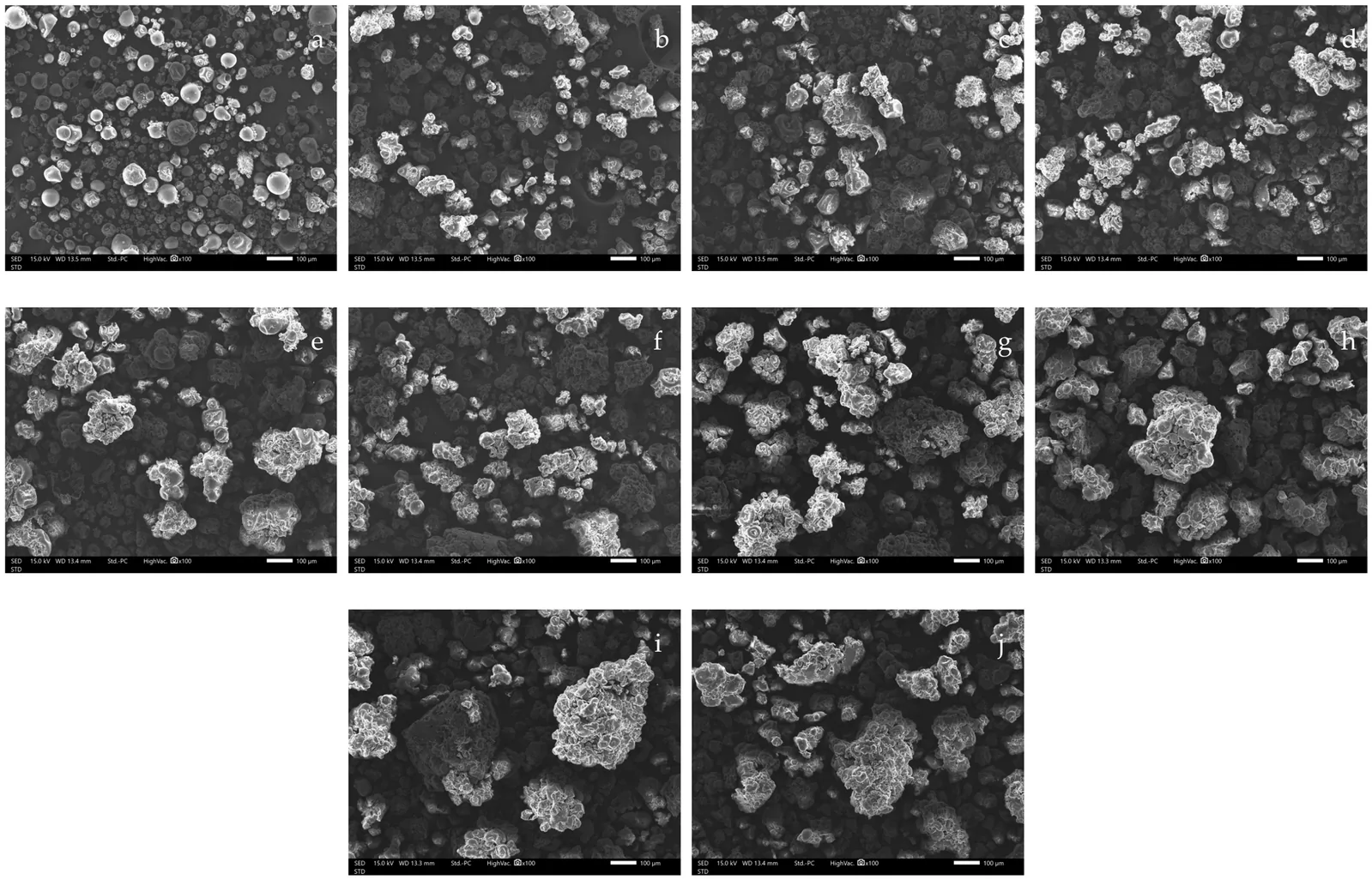
SEM micrographs of SPI formulations; SPI powder (**a**); formulation 1–9 (**b**–**j**).

The SEM micrographs of SPI formulations are shown in Figure 2. The native SPI powder exhibited small, relatively compact, and distinct primary particles with smooth to slightly wrinkled surfaces and spherical-like geometries. This compact and highly cohesive nature was primarily caused by its extremely fine particle size. In typical reconstitution scenarios, these fine, smooth primary particles pack tightly together upon contact with water, minimizing the capillary spaces available for liquid penetration and leading to severe lumping [8]. Following the wet granulation process, the morphological profiles of Formulations 1 through 9 underwent a drastic transformation. The small primary protein particles were successfully agglomerated into significantly larger, irregularly shaped granules. During the wet massing phase, the HPMC solution acted as a liquid binder, forming liquid bridges between the fine SPI particles. Upon drying, these liquid bridges solidified into robust solid bridges, permanently fusing the primary particles into enlarged granular assemblies [36].

The SEM micrographs (Figure 2b–j) confirm the formation of highly porous granules. The restructuring of the dense SPI powder into porous agglomerates is the primary physical mechanism responsible for the drastically improved wettability and dispersing time observed in previous sections. The presence of extensive inter-particulate voids within the granular structure creates a highly efficient capillary network [11]. When these porous granules are introduced to a liquid medium, the internal voids, working synergistically with the potent capillary wicking action of the crospovidone, act as micro-channels that rapidly draw water into the core of the agglomerate. This rapid fluid ingress initiates the immediate swelling and disintegration of the granule into its primary particles before a restrictive outer gel layer can form. Therefore, the morphological shift from fine, dense particles to large, porous granules is directly related to the superior instant-dispersing functionality of the developed formulations, working in tandem with the soluble carbohydrate components of the base matrix.

### 3.4. FTIR

**Figure 3:**
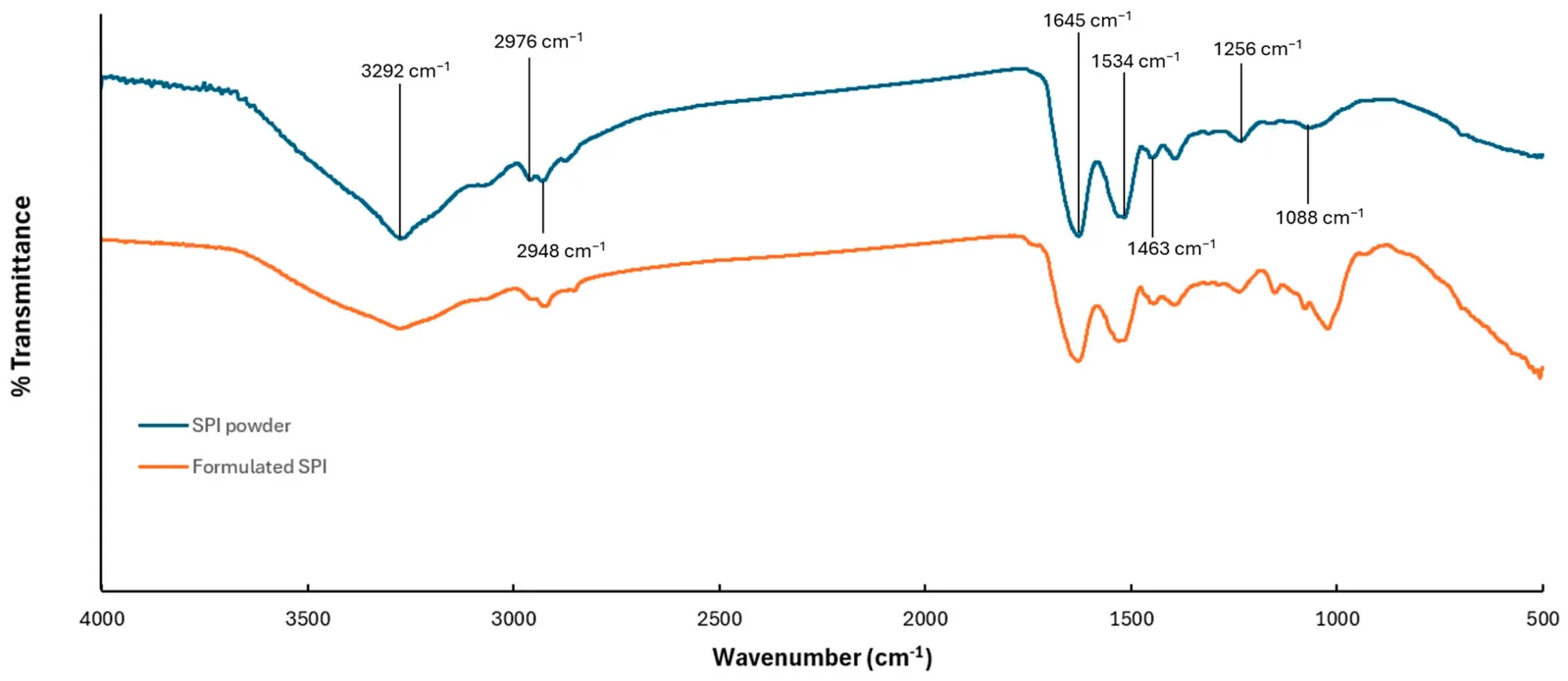
FTIR spectra of SPI powder and formulated SPI.

The FTIR spectra of the native SPI powder and the formulated SPI granules are presented in Figure 3. FTIR spectroscopy provides crucial insights into the molecular structure and chemical bonding of the soy protein isolate. No significant differences were observed in the FTIR spectra among the various formulations. The strong Amide A band at 3292.00 cm^−1^ indicates extensive intermolecular hydrogen bonding within the protein network. The peaks at 2976 and 2948 cm^−1^ correspond to asymmetric and symmetric C–H stretching. The distinct absorption at 1087.68 cm^−1^ points to the minor presence of saccharide structures, a typical characteristic of commercially extracted plant proteins where trace cellular carbohydrates remain associated with the protein matrix [37]. The appearance of the defining Amide I (1645 cm^−1^), Amide II (1534 cm^−1^), and Amide III (1256 cm^−1^) bands confirms the structural integrity of the protein backbone. The Amide I band is particularly sensitive to the secondary structure of proteins.

The practically identical spectral profiles of the raw SPI and the formulated SPI indicate a lack of major chemical alterations. While this visual spectral analysis without amide band deconvolution cannot quantitatively confirm the preservation of specific secondary structures, the absence of significant peak shifts or new signals strongly suggests that the interactions between the soy protein isolate and the polymeric excipients were purely physical. These interactions are primarily driven by non-covalent forces, such as intermolecular hydrogen bonding, rather than chemical cross-linking [37]. Overall, the FTIR spectra confirmed the chemical compatibility within the granular matrix. This ensures that the strategic incorporation of pharmaceutical excipients to enhance macroscopic physical properties such as instant dispersion, superior wettability, and flowability did not compromise or chemically degrade the core nutritional protein structures. Consequently, the wet granulation technique utilizing HPMC and crospovidone was demonstrated to be a structurally safe and chemically inert manufacturing approach for developing plant-based protein supplements.

### 3.5. Moisture Content, Water Activity ($a_{w}$) and pH

Moisture content and water activity ($a_{w}$) are crucial parameters that dictate the physical stability and long-term shelf-life of powdered protein supplements. The moisture content and $a_{w}$ of the SPI formulations are presented in Table 3. The native SPI powder exhibited a moisture content of 6.11 ± 0.17%. Following the wet granulation and subsequent drying processes, the moisture content across all formulations (1–9) was significantly reduced and tightly controlled within the range of 4.35% to 4.87%. Notably, the moisture content of all granulated formulations was successfully maintained below 5%. This reduction from the raw SPI (6.11%) is highly beneficial, as elevated moisture levels can induce the formation of inter-particulate liquid bridges. Such liquid bridges increase the cohesive forces between powder particles, ultimately leading to caking behavior and poor flowability during storage and handling [25,38]. In addition, the moisture content is generally controlled within the range of 3% to 5% to ensure optimum physical stability and flowability [39]. Specifically for plant-based formulations, maintaining moisture levels below 5% is critical to prevent agglomeration and preserve reconstitution properties [40]. In preparation for granulation, the wet granules were dried in a fluid bed granulator. The thermal energy applied during the drying process evaporates not only the moisture from the added binder solution but also the intrinsic free water originally present in the starting raw protein powder. In addition, SPI consists of fine particles (low D0.1 and D0.5 values), which possess a significantly high surface area-to-volume ratio. A larger surface area exposed to the atmosphere facilitates greater moisture adsorption from the environment. In contrast, the granulation process agglomerates these fine particles into larger entities, which drastically reduces the total specific surface area. For these reasons, the formulated granules exhibited a lower moisture content than the raw SPI powder.

The SPI powder exhibited an $a_{w}$ of 0.311 ± 0.033, whereas the $a_{w}$ of all prepared formulations was lower than 0.30. This is significantly lower than the critical threshold of 0.60 required for microbial proliferation [41]. Furthermore, a previous study found that maintaining a low $a_{w}$ is essential for protein isolates to prevent deleterious physical changes, such as the transition from a glassy to a rubbery state, and to inhibit moisture-accelerated chemical degradation, including lipid oxidation and the Maillard browning reaction [42].

The decrease in $a_{w}$ observed in formulations with higher crospovidone and HPMC contents suggests that these hygroscopic excipients effectively interact with and bind the residual free water within the porous granular network. Crospovidone possesses a high capacity for water entrapment via capillary action, thereby minimizing the unbound water available for chemical reactions [14,43]. Consequently, the optimized thermodynamic moisture parameters achieved in these formulations (moisture < 5%, $a_{w}$ < 0.30) are expected to synergistically enhance storage stability and minimize the risk of gelatinous lump formation during reconstitution, addressing a prevalent hydration challenge in plant-based protein powders [25].

While the initial physicochemical characterization demonstrates the success of the granulation process, the long-term physical stability of these formulations remains a critical area for future investigation. The exceptionally low initial moisture content (<5%) and water activity (<0.30) achieved in this study theoretically mitigate the immediate risks of inter-particulate caking [25] and microbial proliferation [41]. However, given the highly hygroscopic nature of both HPMC and crospovidone [14,43], the granular matrix may be susceptible to moisture uptake during prolonged storage. Future studies will focus on evaluating hygroscopicity, caking behavior, and the retention of instant dispersibility over extended 30- and 60-day storage periods under both ambient and accelerated environmental conditions to fully establish the commercial shelf-life of these plant-based protein supplements.

The pH of a protein-based powder formulation is a critical determinant of its solubility, dispersion stability, and overall palatability upon reconstitution. The pH values of the soy protein isolate (SPI) formulations are presented in Table 3. The native SPI powder exhibited a pH of 6.38. Following the wet granulation process and the incorporation of excipients, the pH of all formulations slightly shifted toward a more neutral range (6.52–6.58). Overall, despite the variations in the concentrations of HPMC and crospovidone, all developed formulations successfully maintained a stable, slightly acidic to near-neutral pH suitable for plant-based nutritional beverages. This pH stability indicates that the selected excipients are compatible with the soy protein isolate, avoiding undesired pH-induced protein denaturation. Maintaining this specific pH range is crucial because it is situated well above the isoelectric point (pI) of the major soy protein components (glycinin and *β*-conglycinin), which typically occurs at approximately pH 4.5 [44]. At a pH of approximately 6.5, the protein molecules carry a net negative charge, which induces intermolecular electrostatic repulsion. This electrostatic repulsion prevents protein aggregation and precipitation, thereby enhancing the rehydration capacity, solubility, and phase stability of the protein dispersion when the powder is mixed with water [45].

### 3.6. XRD

**Figure 4:**
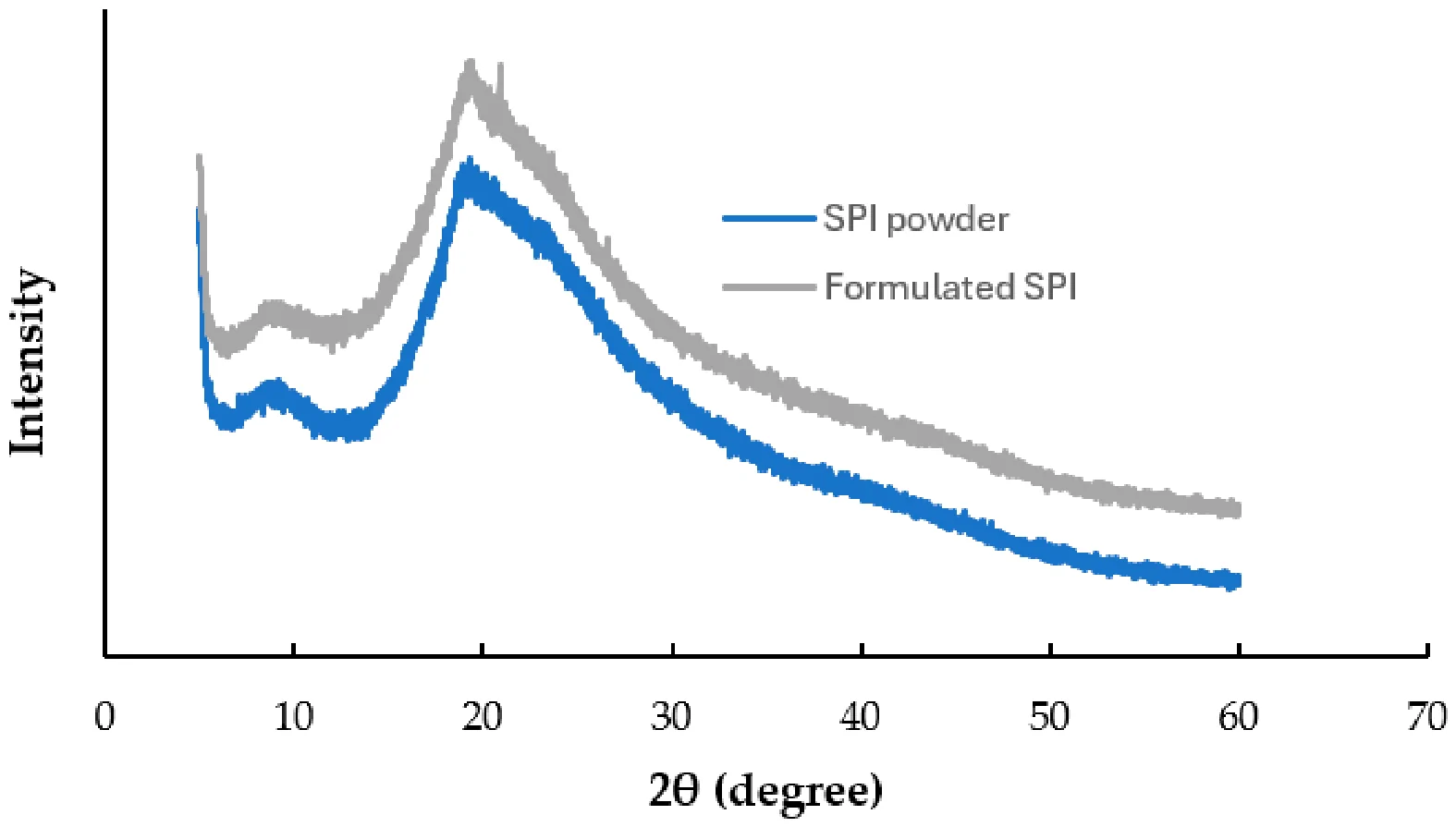
X-ray diffractograms of the SPI powder and formulated SPI.

The X-ray diffractograms of the native SPI powder and the formulated SPI granules are presented in Figure 4. X-ray diffraction was employed to investigate the macroscopic phase state and molecular packing of the protein matrix before and after the formulation process. The absence of sharp diffraction peaks and the presence of broad scattering halos at approximately 2*θ* = 9° and 20° are classic hallmarks of an amorphous material. These broad peaks are attributed to the non-crystalline, randomly coiled macromolecular networks of the 7S and 11S globulins, reflecting the typical *α*-helix and *β*-sheet secondary structural distances within the amorphous protein matrix [46]. The XRD diffractogram of the formulated SPI indicated that the high-shear wet granulation process, including mechanical mixing, hydration with the HPMC binder solution, and subsequent thermal drying, did not induce the formation of new crystalline phases. While the broad amorphous halo cannot definitively prove that the microscopic molecular packing or protein aggregation states remained entirely unchanged, it indicates that the macroscopic amorphous nature of the protein matrix was successfully retained during the process. Furthermore, the pharmaceutical excipients incorporated into the formulations, specifically HPMC and crospovidone, are inherently amorphous polymers [14]. Consequently, their integration into the granular matrix did not contribute to any new crystalline diffraction signals.

### 3.7. DSC

**Figure 5:**
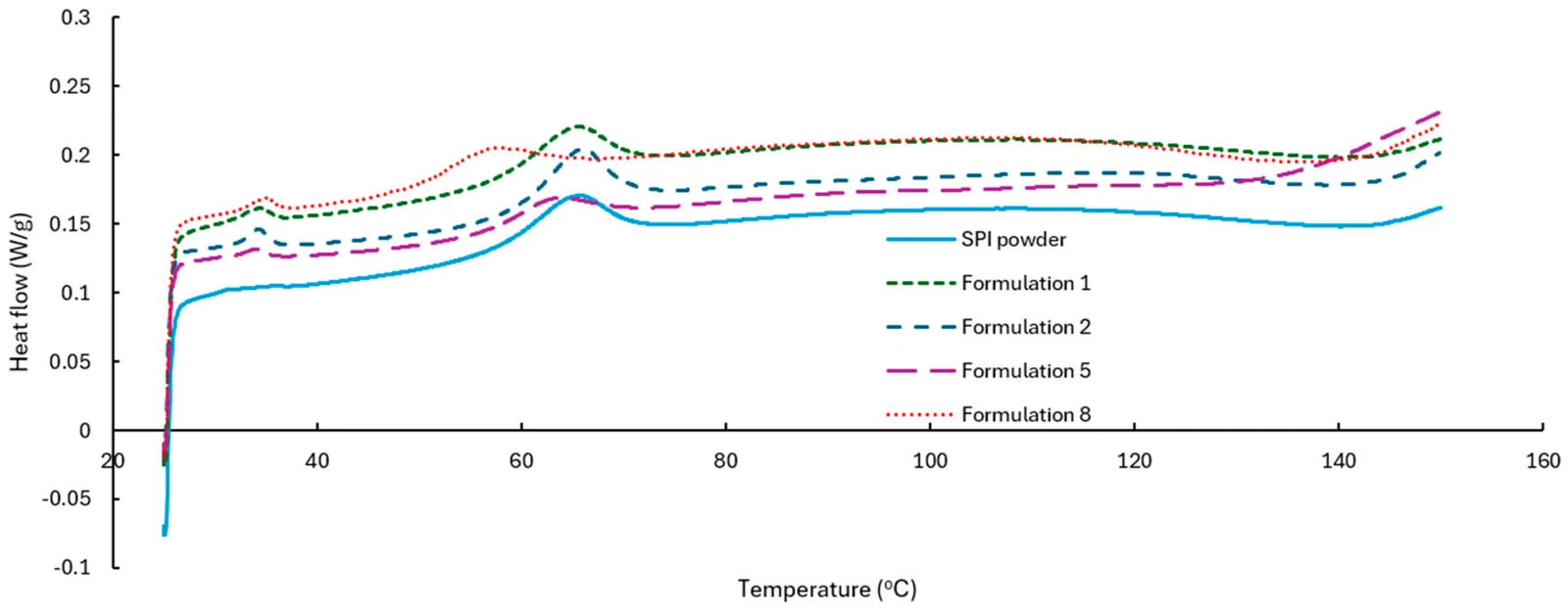
Thermograms of the SPI powder and formulations.

The thermograms of the native SPI powder and the prepared formulations are presented in Figure 5. The thermogram of the unformulated SPI powder displayed a prominent endothermic peak at 67.42 °C, which is characteristic of the thermal denaturation ($T_{d}$) of the 7S globulin (*β*-conglycinin) fraction. While this specific globulin is known for its heat sensitivity, it is important to note that the reported denaturation temperatures ($T_{d}$) for the 7S fraction vary considerably in the literature, generally ranging from approximately 65 °C to over 75 °C [47,48]. These differing results are primarily attributed to variations in extraction methodologies and the specific microenvironmental conditions present during thermal analysis. Beyond the hydration state, the thermal stability and unfolding energy barrier of soy globulins are highly dependent on the pH and ionic strength of the system. Variations in pH alter the net charge of the protein molecules, while ionic strength affects electrostatic shielding, both of which critically dictate the protein’s conformation and intermolecular interactions prior to thermal denaturation. In the granulated formulations, a new minor endothermic peak emerged at low temperatures—specifically at 34.42 °C for formulation 2, 34.08 °C for formulation 5, and 34.92 °C for Formulation 8. This peak, appearing around 34–35 °C, corresponds to the melting point of cocoa butter polymorphs. This lipid component is naturally present in the cocoa powder excipient used to flavor the formulations, which characteristically melts within this narrow physiological temperature range [49]. Furthermore, the $T_{d}$ of the 7S globulin tended to decrease with an increasing proportion of HPMC in the formulations. This shift is primarily driven by the enhanced plasticizing effect induced by higher levels of the hydrophilic polymer during the wet granulation process. When intimately mixed, the HPMC polymeric chains become embedded within the protein matrix. At higher HPMC concentrations, an increased number of hydroxyl groups are introduced into the microenvironment; these disrupt native protein-protein hydrogen bonds and replace them with protein-polymer and protein-water interactions. These excipients, alongside residual moisture, significantly increase the intermolecular free volume and enhance the molecular mobility of the polypeptide chains. Consequently, this pronounced plasticization effect drastically lowers the thermodynamic energy barrier required for the 7S globulin to unfold, leading to a progressively lower denaturation temperature [37].

It should be noted that precise thermodynamic quantification (such as ΔH and strict $T_{d}$ statistics for all formulations) was restricted due to the overlapping complexity of the matrix and potential interference from the prominent cocoa butter melting peak at 34–35 °C. Nonetheless, the observable downward shift in the $T_{d}$ of the 7S globulin indicates a pronounced plasticization effect. While this downward shift indicates a lower thermal unfolding threshold, it is highly advantageous for an instant beverage product. The increased molecular mobility and lower energy barrier inherently facilitate rapid water penetration and accelerated hydration kinetics, directly contributing to the superior wettability and instant-dispersing properties observed in the granulated formulations.

### 3.8. Water Reconstitution

#### 3.8.1. Dispersing Time

Dispersing times of SPI formulations are shown in Table 4. The unformulated SPI powder exhibited a highly prolonged dispersing time exceeding 600 s. Although the wet granulation process reduced the overall dispersing time compared to the raw SPI (>600 s), Formulations 1, 2, and 3 (exhibiting dispersing times of 265.00, 94.33, and 68.33 s, respectively) still failed to meet the <60 s criteria for wettability. This indicates that 0.5% HPMC is insufficient to overcome the hydrophobicity of the matrix. True instant dispersibility was only successfully achieved in formulations with higher excipient concentrations (Formulations 4 to 9). The dispersing time tended to decrease with increasing HPMC and crospovidone fractions in the formulations, especially Formulations 8 and 9, which exhibited significantly shorter dispersing times than others (6.33 ± 0.58 and 5.33 ± 0.58 s, respectively). The raw soy protein isolate’s inability to disperse within 600 s is attributed to its fine particle size and inherent hydrophobicity, which rapidly leads to the formation of a gelatinous, impermeable layer (lump formation) upon initial contact with water [8]. This layer acts as a physical barrier, preventing further liquid penetration into the powder bed.

By agglomerating the fine particles into larger granules, the specific surface area was reduced, and the inter-particulate porosity was increased. According to the principles of capillary action, larger void spaces within the granular network facilitate rapid water ingress, thereby significantly reducing the wetting time [50]. A previous study similarly reported that agglomerating soy protein isolate using a pulsed fluidized bed markedly reduced the wetting time by generating a highly porous granular structure that favored rapid capillary water penetration [11]. The substantial reduction in dispersing time correlated with higher HPMC concentrations indicates that the formation of larger, robust granules facilitates superior capillary action. Larger capillary spaces allow water to penetrate the granules rapidly, preventing the immediate formation of localized viscous blocks on the powder surface [27].

Furthermore, the incorporation of crospovidone acted synergistically to enhance dispersion. Crospovidone functions predominantly through a strong capillary wicking mechanism; its highly porous cross-linked structure draws water deep into the granular matrix rapidly without forming a viscous gel itself [17]. Consequently, formulations containing higher amounts of crospovidone exhibited faster internal hydration and subsequent granular disruption, culminating in the exceptionally swift dispersing times observed in the optimized formulations.

#### 3.8.2. Modified Washburn Method by Capillary Rise Wetting

The water adsorption data for the SPI formulations are presented in Table 4. Water absorption capacity is a crucial indicator of a powder’s ability to imbibe liquid via capillary action, which is a fundamental prerequisite for rapid wetting and dispersion. The raw soy protein isolate powder exhibited the lowest water absorption value of 0.175 ± 0.008 g, indicating poor water absorption due to its highly cohesive nature, small particle size, and lack of internal porosity, which collectively restrict liquid penetration into the powder bed. In granular form, the water absorption capacity significantly increased across all formulations, ranging from 0.185 ± 0.009 g in Formulation 1 to a maximum of 0.382 ± 0.028 g in Formulation 9. The wet granulation process significantly improved this property by transforming the cohesive fine powder into porous agglomerates. This enhanced water absorption is directly linked to the increased inter-particulate void spaces and the structural architecture of the granules, which facilitate water ingress through capillary forces according to the Washburn equation [29].

A clear concentration-dependent relationship was observed with both the polymeric binder and the superdisintegrant. Increasing the HPMC concentration from 0.5% to 1.5% led to a substantial enhancement in water uptake. For example, at a constant 1% crospovidone level, water absorption increased from 0.185 ± 0.009 g (Formulation 1) to 0.339 ± 0.007 g (Formulation 7). Similarly, increasing the crospovidone concentration from 1% to 2% within each HPMC ratio consistently augmented the mass of water absorbed. The highest water absorption capacity was achieved in formulation 9, which incorporated the maximum levels of both HPMC (1.5%) and crospovidone (2%). The progressive increase in water absorption corresponding to higher HPMC concentrations can be attributed to the hydrophilic nature of the polymer matrix. As the HPMC concentration increases, it not only facilitates the formation of larger granules with greater internal void volumes (as observed in the particle size analysis) but also actively hydrates, swells, and retains water within its polymeric network [14]. Ji et al. reported that modifying the physical characteristics of protein-based powders altered their hydration properties, thereby increasing the total mass of water absorbed during the initial reconstitution phase [27].

Furthermore, the incorporation of crospovidone synergistically amplified water uptake. Crospovidone is uniquely characterized by its highly porous, cross-linked structure that relies on a pronounced capillary wicking mechanism to draw large volumes of water rapidly into its matrix without forming a viscous gel barrier [17]. Therefore, the combination of the hydrophilic HPMC framework and the potent wicking action of crospovidone in the optimized formulations (such as Formulation 9) maximized the granular bed’s capacity to absorb water. This elevated water absorption perfectly correlates with the ultra-fast dispersing times observed, as rapid and extensive liquid imbibition is essential for the quick disruption and dissolution of the protein granules.

#### 3.8.3. Dispersibility Index (DI)

The dispersibility index (DI) of SPI formulations is shown in Table 4. The soy protein isolate powder exhibited the highest DI at 33.27 ± 1.03%. This occurs because the raw material consists entirely of fine primary particles; despite exhibiting poor initial wetting and a tendency to form lumps, the fraction of the powder that does enter the water phase can readily pass through the sieve meshes during mechanical stirring.

Following the wet granulation process, the DI values for all formulations decreased. An inverse relationship was clearly observed between the concentration of the HPMC binder and the dispersibility index. Formulations containing the lowest HPMC concentration of 0.5% (Formulations 1–3) maintained the highest DI values among the granules, ranging from 22.14 ± 0.54% to 27.61 ± 2.48%. As the HPMC concentration increased to 1.5% (Formulations 7–9), the DI values dropped significantly to the lowest range of 13.10 ± 1.96% to 15.62 ± 0.87%. The observed reduction in the DI for the granulated formulations reflects the trade-off between improved wetting and granular cohesiveness. Wet granulation intentionally enlarges particle size through the formation of strong solid bridges mediated by the polymeric binder (HPMC). Higher concentrations of HPMC yield a more robust and cohesive granular matrix. Consequently, these robust granules require substantially more time or mechanical shear to completely disintegrate back into primary particles smaller than the test sieve aperture (e.g., 250 µm) within the short testing timeframe [33]. It is important to note that this decrease in DI does not contradict the instant dispersion observed macroscopically. The remarkable instant dispersibility of the optimized formulations is primarily driven by rapid capillary wetting and immediate submersion, which completely eliminates the formation of dry, unwetted lumps on the water surface. While the robust HPMC-bound granules wet and sink instantly (e.g., <10 s), their structural integrity prevents them from fully disintegrating into microscopic primary particles (<250 µm) during the brief 15-s manual stirring window of the DI test. Thus, the lower DI reflects the cohesive strength of the hydrated granular network rather than poor wettability. This robust polymeric network explains the notable decrease in DI as the HPMC concentration increased.

Conversely, escalating the concentration of the superdisintegrant, crospovidone, from 1% to 2% within each respective HPMC tier consistently improved the DI. For example, at a constant 1.0% HPMC level, increasing crospovidone from 1% (Formulation 4) to 2% (Formulation 6) increased the DI from 15.25 ± 1.92% to 20.84 ± 1.46%. As a potent superdisintegrant, crospovidone rapidly draws water into the core of the granules via capillary action and undergoes significant swelling [17]. This rapid swelling exerts internal hydrostatic pressure that actively fractures the polymeric bonds, promoting the breakdown of the granules. This disruptive action perfectly explains the consistent recovery in DI values corresponding to increased crospovidone concentrations across all formulations, highlighting its crucial role in balancing granule structural integrity with necessary dispersion kinetics.

#### 3.8.4. Contact Angle

**Figure 6:**
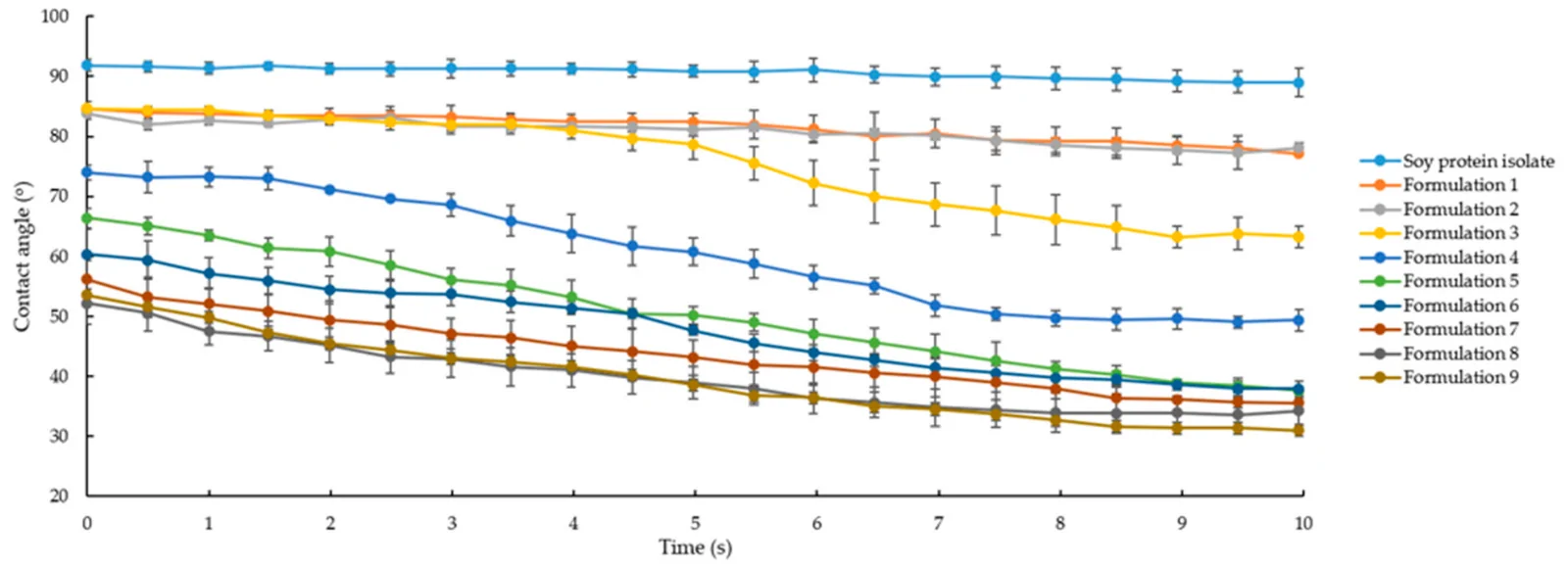
The relationship of contact angle (°) as a function of time using the sessile drop technique.

The contact angle is a primary metric for evaluating the surface wettability of materials. The contact angle data for the SPI formulations and the relationship of the contact angle as a function of time are presented in Table 4 and Figure 6. The dynamic contact angle accurately captures the complex interplay between the initial surface wetting and the subsequent capillary penetration of water into the powder matrix. A contact angle nearing or exceeding 90° generally denotes a hydrophobic surface with poor wettability. The native soy protein isolate exhibited hydrophobicity, demonstrating a high initial contact angle of 91.79 ± 0.98° and an equilibrium angle of 89.46 ± 1.83° (Table 4). The dynamic wetting curve for the raw material remained nearly flat and stable throughout the entire 10 s measurement duration, indicating a strong resistance to water penetration (Figure 2). This behavior is attributed to the outward orientation of hydrophobic amino acid residues, the fine cohesive nature of the powder that entraps air, and the subsequent formation of an impermeable gel layer at the solid–liquid interface, which collectively obstruct water ingress [51].

In contrast, the wet granulation process significantly improved the wettability of the formulations. All granulated formulations (1–9) demonstrated a reduction in both the initial and equilibrium contact angles. The initial contact angle was strongly governed by the concentration of the HPMC binder. Formulations containing 0.5% HPMC (Formulations 1–3) exhibited initial angles ranging from 83.83° to 84.62°, whereas increasing the HPMC concentration to 1.5% (Formulations 7–9) significantly suppressed the initial angles to a range of 52.16° to 56.14° (Table 4). Furthermore, the initial wetting kinetics are not solely dependent on HPMC. The fixed inclusion of maltodextrin (12%), a highly water-soluble carbohydrate, inherently facilitates water ingress, while the lipid content from the cocoa powder (16.33%) introduces localized hydrophobicity. Therefore, the reduction in contact angle and improved capillary action demonstrated by HPMC and crospovidone represent their successful modification of this specific, pre-existing multi-component matrix. The significant drop in the initial contact angle observed in the granulated formulations can be directly correlated with the incorporation of HPMC. As a highly hydrophilic polymer, HPMC effectively coats the surface of the cohesive soy protein particles during the wet massing phase. Upon drying, this hydrophilic polymeric film drastically lowers the solid–liquid interfacial tension when a water droplet is introduced, promoting immediate initial wetting and spreading over the granular bed surface [13]. This phenomenon explains why formulations with 1.5% HPMC exhibited substantially lower initial contact angles compared to those with 0.5% HPMC; a higher binder concentration ensures a more comprehensive hydrophilic surface coverage, which is a critical attribute for accelerating the dynamic wetting kinetics of poorly wettable powders [15]. A previous study similarly reported that the use of a hydrophilic polymer such as HPMC as a binder decreased the surface tension and contact angle of water-insoluble or hydrophobic powders [13].

Furthermore, the rate at which the contact angle decayed toward equilibrium was highly dependent on the superdisintegrant concentration. Formulations with higher crospovidone levels, particularly within the 1.5% HPMC tier, exhibited a steep and immediate decline in their dynamic contact angle curves. Formulation 9, containing the maximum amounts of both excipients, achieved the lowest equilibrium contact angle of 32.08 ± 1.58° within the 10 s timeframe. The steep decline in the contact angle curves, particularly for formulations with 2% crospovidone (e.g., Formulations 6 and 9), highlights the potent capillary wicking action of the superdisintegrant. Crospovidone’s highly porous, cross-linked structure rapidly draws the water droplet from the surface into the internal voids of the granule [17,18]. This rapid internal hydration perfectly mirrors the previously observed enhancements in water absorption and dispersing time. Synergistically, the hydrophilic HPMC facilitates immediate surface spreading, while the crospovidone drives rapid internal wicking, effectively transforming the hydrophobic soy protein into a highly wettable and instantly dispersible granular matrix.

However, in the sessile drop method, the samples were compressed before testing because the water rapidly penetrated through the samples. To confirm the contact angle of the granular samples, the contact angles were further evaluated using the modified Washburn capillary rise method, and the results are presented in Table 4. The native SPI powder exhibited a significantly higher contact angle of 84.12 ± 1.64° (*p* < 0.05), attributable to the strong hydrophobic nature and poor water affinity of the native protein matrix. Upon granulation, the contact angles decreased with increasing concentrations of HPMC and crospovidone. The formulations exhibited a steady decline from 76.70 ± 0.95° in Formulation 1 down to the significantly lowest value of 43.86 ± 2.20° in Formulation 9 (*p* < 0.05). Notably, there were no significant differences between Formulations 1 and 2, as well as between Formulations 7 and 8 (*p* > 0.05), indicating specific concentration thresholds where the capillary wetting behavior stabilizes before improving further at higher excipient levels. This significant reduction in the contact angle provides quantitative evidence of enhanced capillary action within the true granular state. While the highly water-soluble carbohydrate components in the fixed base matrix (such as maltodextrin) provide a baseline for water ingress, the progressive improvement observed across Formulations 1 to 9 highlights the successful modifying roles of HPMC and crospovidone. HPMC effectively lowers the solid–liquid interfacial tension upon initial contact, while the highly porous granular structure, supported by crospovidone acting as an insoluble physical water conduit, facilitates rapid liquid penetration deep into the powder bed. Furthermore, these Washburn contact angle results exhibit a strong quantitative correlation with the macroscopic dispersing times and water adsorption capacities. As the dynamic contact angle of the granular bed decreased, the ability of the liquid to rapidly wick into the inter-particulate voids drastically improved, directly contributing to the ultra-fast submersion time of 5.33 s achieved in Formulation 9.

It is important to note that the physical, thermal, and rheological behaviors observed across Formulations 1–9 are the collective result of the entire complex matrix, which includes fixed concentrations of cocoa powder, xanthan gum, and carrageenan. While the experimental design did not include blank granulation controls (0% HPMC or 0% crospovidone) to prove statistical synergism against the base matrix, the results clearly demonstrate that varying the concentrations of HPMC and crospovidone served as critical modifying factors. Within this pre-established formulation, these two excipients successfully manipulated the dispersion kinetics and capillary water uptake to achieve instant dispersibility.

### 3.9. Apparent Viscosity

The rheological behavior of reconstituted protein beverages is a critical attribute that dictates not only physical stability but also sensory perception, such as mouthfeel and texture. The shear-thinning (pseudoplastic) behavior observed in all granulated formulations is highly desirable for liquid nutritional products. At low shear rates (e.g., during storage or resting in a glass), the high apparent viscosity helps suspend insoluble particles and prevents phase separation. Conversely, at high shear rates (e.g., during swallowing or mastication), the viscosity drops significantly, providing a smooth, non-slimy mouthfeel.

**Figure 7:**
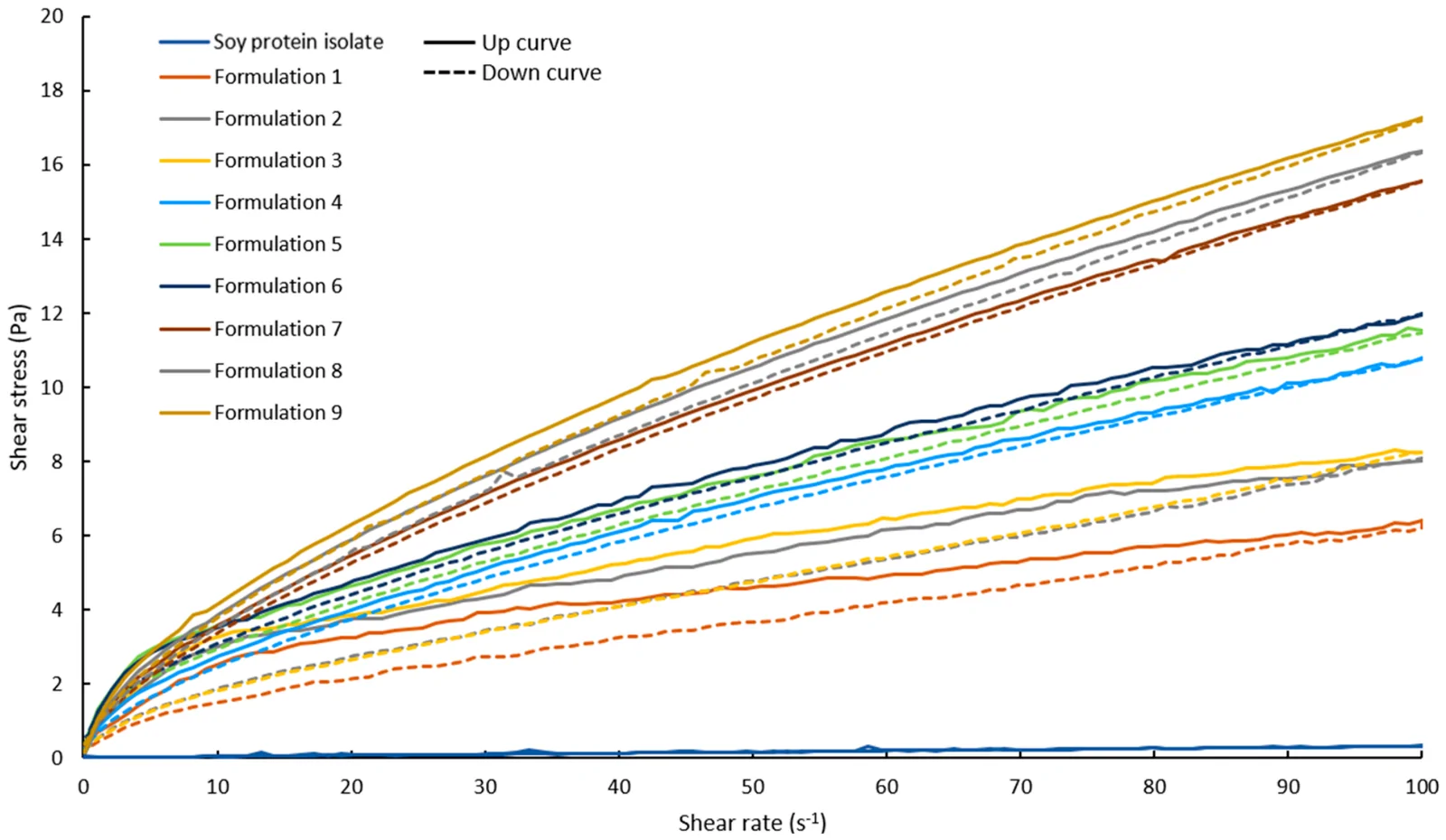
Rheological behaviors of SPI formulations.

The rheological behavior of SPI formulations is presented in Figure 7. The native SPI exhibited Newtonian flow behavior, characterized by extremely low and relatively constant shear stress across the tested shear rate range. In contrast, all formulations demonstrated a distinct pseudoplastic (shear-thinning) behavior, where the shear stress increased at a decreasing rate as the shear rate increased. This shear-thinning phenomenon occurs because, at rest, the hydrated macromolecular chains of HPMC and the unfolded soy protein molecules form a randomly entangled, complex three-dimensional network. Previous studies have reported that dispersions of globular plant proteins and long-chain polysaccharides typically exhibit pseudoplasticity due to the reversible alignment and disentanglement of polymer coils under shear stress [16,52]. When shear stress is applied, these long polymeric chains align themselves in the direction of the flow, thereby reducing internal flow resistance and causing a sharp drop in apparent viscosity [16]. Marcotte et al. found that higher HPMC concentrations exponentially increase the viscosity of the continuous aqueous phase by forming a denser, intertwined polymeric network [16]. It is crucial to emphasize that this fundamental shear-thinning behavior is primarily governed by the fixed hydrocolloid base matrix of the formulations, specifically xanthan gum (1%) and carrageenan (1%). Upon hydration, these hydrocolloids form a robust, entangled three-dimensional network that aligns along the direction of flow under applied shear, leading to the observed reduction in apparent viscosity. Varying the concentrations of HPMC and crospovidone acted as significant modifying factors within this matrix. Formulations with higher HPMC concentrations exhibited higher shear stress values across all shear rates compared to those with lower concentrations. This indicates that the hydrated macromolecular chains of HPMC interpenetrated and reinforced the existing hydrocolloid network, thereby increasing the overall flow resistance. Furthermore, an analysis of the upward and downward continuous shear curves reveals a distinct hysteresis loop across Formulations 1–9, where the downward curves consistently present lower shear stress values than their corresponding upward curves. This hysteresis confirms the presence of thixotropic behavior, indicating a time-dependent structural breakdown of the fluid network under continuous shear and a subsequent gradual recovery when the shear is reduced. Notably, the hysteresis area tended to broaden in formulations with higher HPMC concentrations (Formulations 7–9), reflecting a more complex and densely entangled microstructural network.

To further quantify these rheological profiles, the experimental data were fitted to the Power-law model, and the parameters are presented in Table 5. The model demonstrated an excellent fit for all samples, showing high determination coefficients ($R^{2}>0.96$). The flow behavior index (*n*) for all samples was less than 1, mathematically confirming their pseudoplastic nature. The native SPI powder exhibited a nearly Newtonian-like behavior, with the highest *n* value of 0.8648 and the lowest consistency coefficient (*K*) of 0.017523 Pa·s. The incorporation of the HPMC binder significantly altered the flow dynamics. Formulations containing the highest HPMC concentration (1.5%, Formulations 7–9) exhibited the most prominent shear-thinning behavior, marked by the lowest *n* values (ranging from 0.4415 to 0.4633). The increase in HPMC concentration tended to increase the consistency coefficient (*K*). For example, the *K* value increased from 0.126999 Pa·s in Formulation 1 (0.5% HPMC) to a maximum of 0.672048 Pa·s in Formulation 9 (1.5% HPMC). Beyond physical stability, these rheological profiles hold significant sensory implications for the target demographic. The pseudoplastic behavior imparted by HPMC provides a smooth, lubricating mouthfeel during swallowing, which is highly beneficial for elderly or dysphagic individuals [53,54]. Furthermore, potential concerns regarding grittiness introduced by insoluble excipients, such as crospovidone, are effectively mitigated by the primary particle size dynamics of the formulation. Once the granules completely disintegrate in water, the resulting primary SPI particles and the crospovidone particles (which possess a fine primary size of 5–50 µm) fall well below the typical human oral detection threshold for grittiness (generally >50 µm) [55,56]. This size profile ensures that the reconstituted beverage maintains a palatable, smooth texture without perceptible chalkiness or grittiness.

The Power-law parameters further elucidate the structural impact of formulation components. It is crucial to emphasize that the baseline shear-thinning behavior and the overall viscosity observed across all granulated formulations are primarily governed by the fixed presence of xanthan gum (1%) and carrageenan (1%) in the base matrix. These potent hydrocolloids naturally form a strong, entangled three-dimensional network upon hydration. Within this established complex matrix, the addition of the HPMC binder acts as an additional modifying factor. As the HPMC concentration increases, its hydrated macromolecular chains interpenetrate with the existing hydrocolloid network, further thickening the continuous aqueous phase and contributing to the higher consistency coefficients (*K*) observed in Formulations 7–9.

Interestingly, while elevated viscosity generally impedes liquid penetration and exacerbates the formation of undispersed lumps in conventional protein powders [8], this study reveals a synergistic formulation effect. Specifically, Formulation 9, which possesses the highest consistency coefficient, simultaneously demonstrated the fastest dispersing time and the lowest contact angle. This seemingly contradictory outcome can be explained by the potent capillary wicking action of crospovidone [17]. The superdisintegrant draws water into the granular core so rapidly that it fractures the granule structure before the outer HPMC layer can fully hydrate to form a highly viscous, impermeable gel barrier. Once the granules are successfully shattered into primary particles, the HPMC then hydrates completely to provide the targeted high consistency. This highlights that the strategic combination of crospovidone and HPMC successfully decouples the initial wetting/dispersing properties from the final rheological properties, allowing for the creation of an instantly dispersible protein powder while providing a viscous beverage upon complete reconstitution.

### 3.10. Protein Content

The protein content of SPI formulations is shown in Table 6. The reduction in the overall protein percentage of the granulated formulations compared to the raw material is an expected outcome of the formulation design, primarily due to the dilution effect of the added excipients. As detailed in the formulation compositions (Table 1), the raw soy protein isolate powder constitutes between 76.07% and 78.07% of the total mass in the final products. Thus, the protein content in formulations should range from 66.74% to 68.08%. These results confirmed that the wet granulation process did not cause any unexpected degradation or loss of protein. In addition, the selected excipient ratios and the manufacturing process successfully maintained a consistent, high-protein macronutrient profile across all optimized formulations, making them highly suitable for plant-based nutritional supplements.

## 4. Conclusions

This study successfully demonstrated a highly effective and novel multidisciplinary approach by applying pharmaceutical-grade excipients (HPMC and crospovidone) via high-shear wet granulation to resolve the inherent dispersion limitations of soy protein isolate (SPI). The granulation process structurally transformed the fine, hydrophobic, and cohesive SPI powder into large, highly porous agglomerates. The combined incorporation of HPMC, acting as a hydrophilic coating binder, and crospovidone, providing aggressive capillary wicking, drastically improved the macroscopic reconstitution properties. Within the fixed beverage matrix, the optimized formulation (incorporating 1.5% HPMC and 2.0% crospovidone) achieved a fast dispersing time of 5.33 s, a substantial increase in water absorption, and a significantly reduced equilibrium contact angle (32.08°), completely overcoming the problematic lumping phenomenon.

Furthermore, structural evaluations through FTIR and XRD confirmed that the granulation process induced purely physical interactions without compromising the chemical integrity, native functional groups, or the amorphous nature of the protein. Thermal analysis revealed a beneficial, concentration-dependent plasticization effect that lowered the unfolding energy barrier, further aiding rapid hydration. Additionally, the reconstituted formulations exhibited highly favorable shear-thinning (pseudoplastic) rheological behavior.

From an industrial perspective, the wet granulation process coupled with fluid-bed drying offers excellent scalability for plant protein powder manufacturing. Unlike conventional spray drying, which requires exceptionally high inlet temperatures (often exceeding 150 °C) that risk severe and irreversible thermal denaturation of the heat-sensitive 7S and 11S globulins, the wet granulation process operates under notably mild thermal conditions. This controlled thermal environment significantly minimizes the risk of structural degradation during large-scale processing, ensuring that the functional, nutritional, and conformational integrity of the plant-based protein is preserved in industrial manufacturing.

In conclusion, the wet granulation process utilizing HPMC and crospovidone effectively modified the complex SPI matrix to significantly improve its macroscopic dispersibility in water. This formulation strategy presented a safe, scalable, and chemically inert manufacturing solution to resolve the fundamental dispersion limitations of soy protein matrices. While the initial physicochemical properties demonstrate significant technological potential for developing convenient nutritional formulations for the elderly, future comprehensive studies are required. Extended evaluations focusing on long-term storage stability (including moisture sorption and particle degradation), sensory acceptability, mouthfeel, and in vitro digestibility are essential to fully validate its clinical suitability and commercial viability as a plant-based protein supplement for sarcopenic populations.

## Figures and Tables

**Table 1:** Soy protein isolate formulation.

No.	Composition (% *w*/*w*)									Total
	Soy Protein Isolate	3% HPMC Solution	Crosprovidone	Sucralose	Maltodextrin	Sodium Saccharin	Xanthan Gum	Carrageenan	Cocoa Powder	
1	78.07	0.5	1	0.05	12	0.05	1	1	6.33	100
2	77.57	0.5	1.5	0.05	12	0.05	1	1	6.33	100
3	77.07	0.5	2	0.05	12	0.05	1	1	6.33	100
4	77.57	1	1	0.05	12	0.05	1	1	6.33	100
5	77.07	1	1.5	0.05	12	0.05	1	1	6.33	100
6	76.57	1	2	0.05	12	0.05	1	1	6.33	100
7	77.07	1.5	1	0.05	12	0.05	1	1	6.33	100
8	76.57	1.5	1.5	0.05	12	0.05	1	1	6.33	100
9	76.07	1.5	2	0.05	12	0.05	1	1	6.33	100

**Table 2:** Particle size distribution of SPI formulations.

Formulation	D (0.1) (µm)	D (0.5) (µm)	D (0.9) (µm)	D [3, 2] (µm)	D [4, 3] (µm)	Span
SPI powder	23.92 ± 0.57 ^a^	59.66 ± 1.82 ^a^	1195.42 ± 301.76 ^a^	54.71 ± 1.51 ^a^	280.0 ± 40.64 ^a^	19.55 ± 4.43 ^a^
1	27.16 ± 0.37 ^b^	71.86 ± 1.66 ^b^	211.44 ± 17.55 ^b^	50.23 ± 1.04 ^b^	102.19 ± 5.55 ^bc^	2.29 ± 0.19 ^b^
2	31.36 ± 0.38 ^c^	78.46 ± 1.01 ^b^	222.71 ± 27.19 ^bc^	57.19 ± 0.88 ^c^	118.60 ± 7.53 ^bc^	2.72 ± 0.91 ^bcd^
3	32.83 ± 0.42 ^c^	88.33 ± 5.17 ^c^	328.46 ± 70.89 ^c^	63.02 ± 2.71 ^d^	139.29 ± 19.44 ^c^	3.20 ± 0.20 ^c^
4	46.93 ± 1.48 ^d^	135.07 ± 13.95 ^d^	478.86 ± 88.56 ^d^	93.82 ± 6.27 ^e^	218.51 ± 16.32 ^d^	3.18 ± 0.33 ^cd^
5	53.85 ± 0.23 ^e^	163.67 ± 3.71 ^e^	656.61 ± 18.18 ^ef^	115.27 ± 0.86 ^f^	271.66 ± 3.30 ^d^	3.69 ± 0.19 ^d^
6	49.91 ± 0.09 ^f^	134.79 ± 1.73 ^d^	508.40 ± 19.15 ^d^	100.64 ± 0.71 ^e^	216.86 ± 6.27 ^d^	3.40 ± 0.13 ^cd^
7	66.26 ± 0.58 ^g^	180.76 ± 4.07 ^f^	622.44 ± 20.40 ^ef^	136.82 ± 1.90 ^g^	271.65 ± 7.68 ^ad^	3.08 ± 0.08 ^c^
8	56.48 ± 0.21 ^h^	152.42 ± 3.17 ^d^	543.56 ± 11.14 ^df^	113.31 ± 1.15 ^f^	235.14 ± 4.09 ^ad^	3.02 ± 0.05 ^c^
9	62.45 ± 0.91 ^i^	176.09 ± 9.64 ^f^	610.00 ± 69.67 ^ef^	126.61 ± 4.73 ^h^	265.71 ± 24.28 ^ad^	3.10 ± 0.26 ^c^

Different superscript letters in each column indicate significant differences (*p* < 0.05).

**Table 3:** Moisture content, water activity and pH of SPI formulations.

Formulation	Moisture Content (%)	Water Activity	pH
SPI powder	6.11 ± 0.17 ^a^	0.311 ± 0.033 ^a^	6.38 ± 0.03 ^a^
1	3.54 ± 0.16 ^b^	0.299 ± 0.003 ^a^	6.59 ± 0.05 ^b^
2	3.35 ± 0.30 ^b^	0.267 ± 0.003 ^ab^	6.52 ± 0.02 ^b^
3	3.69 ± 0.27 ^b^	0.199 ± 0.018 ^c^	6.56 ± 0.06 ^b^
4	3.65 ± 0.09 ^b^	0.203 ± 0.013 ^c^	6.53 ± 0.04 ^b^
5	3.68 ± 0.15 ^b^	0.203 ± 0.013 ^c^	6.56 ± 0.01 ^b^
6	3.73 ± 0.15 ^b^	0.183 ± 0.021 ^c^	6.58 ± 0.05 ^b^
7	3.87 ± 0.06 ^b^	0.150 ± 0.005 ^d^	6.56 ± 0.03 ^b^
8	3.85 ± 0.10 ^b^	0.184 ± 0.026 ^cd^	6.56 ± 0.01 ^b^
9	3.82 ± 0.09 ^b^	0.126 ± 0.009 ^e^	6.57 ± 0.02 ^b^

Different superscript letters in each column indicate significant differences (*p* < 0.05).

**Table 4:** Water reconstitution properties of SPI formulations.

Formulation	Dispersing Time (s)	Water Adsorption (g)	Dispersibility Index (DI)	Contact Angle (°) by Sessile Drop		Contact Angle (°) by Modified Washburn Capillary Rise
				Initial (°)	Equilibrium (°)	
SPI powder	>600	0.175 ± 0.008 ^a^	33.27 ± 1.03 ^a^	91.79 ± 0.98 ^a^	89.46 ± 1.83 ^a^	84.12 ± 1.64 ^a^
1	265.00 ± 91.65 ^a^	0.185 ± 0.009 ^ac^	22.14 ± 0.54 ^b^	84.56 ± 1.25 ^b^	78.84 ± 1.65 ^b^	76.70 ± 0.95 ^b^
2	94.33 ± 37.90 ^b^	0.239 ± 0.009 ^cd^	25.09 ± 3.43 ^bc^	83.83 ± 1.00 ^b^	78.18 ± 1.89 ^b^	76.72 ± 1.19 ^b^
3	68.33 ± 14.22 ^b^	0.271 ± 0.031 ^de^	27.61 ± 2.48 ^c^	84.62 ± 0.45 ^b^	65.12 ± 3.11 ^c^	71.89 ± 1.44 ^c^
4	12.33 ± 0.58 ^c^	0.298 ± 0.032 ^def^	15.25 ± 1.92 ^df^	73.95 ± 1.18 ^c^	49.64 ± 1.30 ^d^	68.69 ± 1.47 ^cd^
5	9.67 ± 0.58 ^de^	0.328 ± 0.037 ^efg^	18.25 ± 0.55 ^e^	66.53 ± 1.63 ^d^	40.38 ± 1.47 ^e^	67.87 ± 0.75 ^d^
6	8.33 ± 0.58 ^d^	0.338 ± 0.007 ^fg^	20.84 ± 1.46 ^e^	60.34 ± 4.20 ^e^	39.25 ± 1.37 ^ef^	65.39 ± 0.88 ^e^
7	10.33 ± 0.58 ^e^	0.339 ± 0.007 ^fg^	13.10 ± 1.96 ^d^	56.14 ± 3.12 ^f^	37.02 ± 1.44 ^f^	57.15 ± 1.35 ^f^
8	6.33 ± 0.58 ^f^	0.373 ± 0.003 ^g^	15.62 ± 0.87 ^dg^	52.16 ± 3.54 ^f^	33.96 ± 2.72 ^g^	51.65 ± 5.02 ^f^
9	5.33 ± 0.58 ^f^	0.382 ± 0.028 ^g^	18.15 ± 1.37 ^efg^	53.52 ± 0.49 ^f^	32.08 ± 1.58 ^g^	43.86 ± 2.20 ^g^

Different superscript letters in each column indicate significant differences (*p* < 0.05).

**Table 5:** Parameters from Power-law model.

Formulations	Flow Behavior Index (*n*)	Consistency Coefficient (Pa.s)	R^2^
SPI powder	0.8648	0.017523	0.9949
1	0.5456	0.126999	0.9708
2	0.597	0.102117	0.9821
3	0.6019	0.084625	0.9731
4	0.5492	0.248028	0.9808
5	0.577	0.19081	0.9703
6	0.52	0.222433	0.9711
7	0.4633	0.292483	0.9696
8	0.4515	0.577697	0.9702
9	0.4415	0.672048	0.9759

**Table 6:** Protein content of SPI formulations.

Formulations	Protein Content (%)
SPI powder	86.10 ± 3.54 ^a^
1	67.78 ± 2.45 ^b^
2	67.04 ± 3.61 ^b^
3	66.74 ± 2.87 ^b^
4	67.92 ± 1.98 ^b^
5	67.67 ± 2.14 ^b^
6	67.24 ± 3.28 ^b^
7	68.08 ± 1.39 ^b^
8	67.61 ± 2.06 ^b^
9	66.97 ± 2.57 ^b^

Different superscript letters in each column indicate significant differences (*p* < 0.05).

## Data Availability

The original contributions presented in this study are included in the article. Further inquiries can be directed to the corresponding author.

## Abbreviations

The following abbreviations are used in this manuscript:

HPMC	Hydroxypropyl methylcellulose
SPI	Soy protein isolate
